# The Phenomenon of Compensatory Cell Proliferation in Olfactory Epithelium in Fish Caused by Prolonged Exposure to Natural Odorants

**DOI:** 10.1038/s41598-020-65854-9

**Published:** 2020-06-01

**Authors:** Igor V. Klimenkov, Nikolay P. Sudakov, Mikhail V. Pastukhov, Nikolay S. Kositsyn

**Affiliations:** 1 Limnological Institute, Siberian Branch, Russian Academy of Sciences, 3 Ulan-Batorskaya St., Irkutsk, 664033 Russia; 2 Irkutsk State University, 1 Karl Marx St., Irkutsk, 664003 Russia; 3 Vinogradov Institute of Geochemistry, Siberian Branch, Russian Academy of Sciences, 1a Favorsky St., Irkutsk, 664033 Russia; 4 Institute of Higher Nervous Activity and Neurophysiology, Russian Academy of Sciences, 5a Butlerova St., Moscow, 117485 Russia

**Keywords:** Cellular neuroscience, Adult neurogenesis

## Abstract

It was previously shown that activation of the processes of neurogenesis in the olfactory epithelium (OE) can be caused after intranasal administration of toxic or neurotrophic factors, after axon transection, or as a result of bulbectomy. Our study showed for the first time that a significant increase in olfactory cell renewal can also occur in animals due to periodic chemostimulation with natural odorants (amino acids and peptides) for 15 days. Using electron and laser confocal microscopy in fish (*Paracottus knerii* (Cottidae), Dybowski, 1874) from Lake Baikal, we showed that periodic stimulation of aquatic organisms with a water-soluble mixture of amino acids and peptides causes stress in OE, which leads to programmed death cells and compensatory intensification of their renewal. We estimated the level of reactive oxygen species, number of functionally active mitochondria, intensity of apoptosis processes, and mitosis activity of cells in the OE of fish in the control group and after periodic natural odorants exposure. This study showed that new stem cells are activated during enhanced odor stimulation and subsequent degenerative changes in the cells of the sensory apparatus. Those new activated stem cells are located in previously proliferatively inactive regions of OE that become involved in compensatory processes for the formation of new cells.

## Introduction

Neurogenesis in the olfactory epithelium (OE) of animals is maintained throughout their life due to the proliferative activity of regional stem cells (SC) that produce various types of cells in OE^1–4^. The regeneration ability is prospective for the OE to serve as a source of SC for detailed study and therapeutic use^4^. Model experiments indicate that various forms of sensory deprivation can significantly activate the natural rate of neurogenesis inherent for intact animals. Thus, after destructive changes in the epithelium resulted from exposure to toxic agents, axon transection or bulbectomy, cell proliferation increases^5–11^. Moreover, olfactory neurogenesis can also be activated by intranasal administration of neurotrophic factors^12^. Considering the prospective use of these technologies in medicine, new information on the mechanisms of olfactory neurogenesis is mainly intensively studied in mammals. In this regard, fish have been studied in less detail. However, the cytological and molecular mechanisms lying at the root of olfactory neurogenesis in fish may also be of interest for the study of neuroplasticity and for development of treatment methods for neurodegenerative diseases^13–15^.

It has been established that olfactory neurogenesis processes in fish can also be activated with similar methods of sensory deprivation^16–21^. It should be noted that all these methods for activating neurogenesis are mostly invasive and traumatizing, which limits their application in practical medicine.

In this publication we present a new approach for stimulating of cell proliferation in OE, which allows one to avoid these complications. In order to activate this processes, we proceeded from the two main facts: (1) amino acids (and, to a lesser extent, peptides) and their mixtures are effective natural odorants for fishes^22–27^, and (2) OSNs are monospecific^28–30^, which is why we used a mixture of such structurally diverse molecules to increase the number of odorant-stimulated OSNs. We applied a long (15 days) periodic (12 hours every day) exposure of OE in fish to mixture of a wide range of natural water-soluble odors (amino acids and peptides). This effect caused stress in a large amount of OSN, which was accompanied by degenerative changes in the mitochondria and activation of programmed cell death (PCD), which led to a compensatory increase in the activity of mitotic stem cells (SC) in OE.

## Results

Based on structural features, spatial arrangement, and specifics of molecular markers, in fish five types of OSNs were identified, which enable the perception of different types of odorants: ciliated OSNs, microvillous OSNs, crypt neurons, pear-shaped OSNs, and kappe neurons^31–34^. Ciliated and microvillous cells are the most numerous cells, that are sensitive to amino acids and taurocholic acid^25,35^.

The other types of OSNs are minor in terms of quantity; however, they have functional differences. Crypt neurons perceive bile acids, pheromonal signals, and amino acids^36–38^, while pear-shaped OSNs specialize in the reception of nucleotides, which arrive from nutritional sources into the aqueous medium^34^. The functional specialization of kappe neurons has not been determined yet.

**Figure 1:**
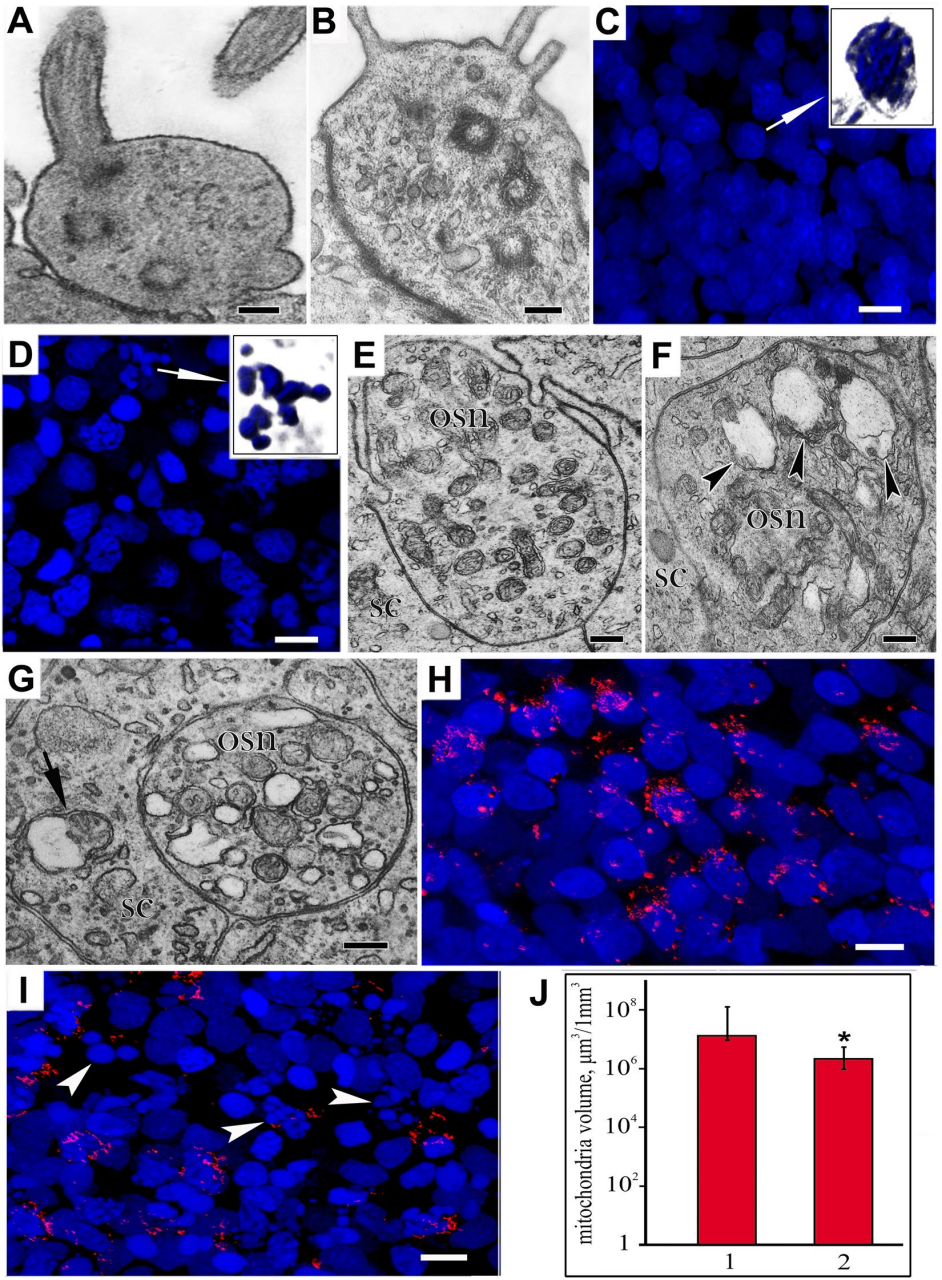
Cytochemical features of the OE in *P. knerii* in the control group and after prolonged periodic odorant stimulation. The apex of a ciliated cell (**A**) with a cilium and a microvillar cell (**В**) with a few microvilli; transmission electron microscopy. The nuclei of OE cells in the control group (**С**) and the experimental group (**D**), in which the nuclei (indicated by arrows) of the individual cells exhibited signs of PCD. On the insets: a separate nucleus in the control (**C**) and apoptotic bodies in the experiment (**D**); DAPI staining (blue); confocal microscopy. (**E**) Slice of dendrite with mitochondria in control; transmission electron microscopy. (**F**) Degenerative changes in mitochondria (indicated by arrows) in an OSN dendrite; transmission electron microscopy. (**G**) Degenerative changes in mitochondria (indicated by arrows) in OSN and SC; transmission electron microscopy. (**H**) Functionally active mitochondria in the OE of a control fish (MitoTracker Orange staining, red) and after prolonged odorant exposure. (**I**) The arrows indicate the numerous apoptotic bodies; confocal microscopy. (**J**) The volume occupied by the fluorescent signal of functionally active mitochondria in the control group (1) and after prolonged odorant chemostimulation (2). The graph shows the data obtained through the quantitative analysis of the Z-stacks; confocal microscopy. *p_u_ ≤ 0.05 compared with the control group. Notation: OSN – olfactory sensor neuron; SC – supporting cell. Scale bars: A, B, 0,25 mkm; C, D, 5 mkm; E‒G, 0,5 mkm; H, I, 5 mkm.

This study was conducted on *Paracottus knerii* (Cottidae) (Dybowski, 1874) (Supplementary Fig. 1), an endemic representative of the ichthyofauna of Lake Baikal. In *P. knerii*, the olfactory rosette is 2–2.5 mm in size and, as in other Baikal Cottoidei, has 5–6 folds (Supplementary Fig. 2). The OE has a structure typical of that of teleosts^39,40^ and other Cottidae^41,42^ and consists of receptor, supporting, and basal cells. Based on transmission microscopy data, the OE mainly contains two types of receptor cells, namely, ciliated and microvillar receptor cells (Fig. 1A,B). It was established that, compared with that of the control (Fig. 1C), the OEs of the experimental fish, which were subjected to intensive odorant exposure to a mixture of amino acids and peptides, showed signs of densification and fragmentation of nuclei in epithelial cellular elements, which serve as an indicator of the final phase of PCD^43^. The nuclei of these cells looked like fragmented round formations 0.5 to 3 µm in size with condensed, intensely DAPI-stained chromatin (Fig. 1D). In 10–15% of receptor neurons, endoplasmic reticulum channels looked fragmented and, in some areas, widened, and dendrites contained mitochondria with distinct signs of partial or complete swelling. Compared with that in the control (Fig. 1E), these organelles had a clear matrix with loose electron-dense deposits and destructive changes in the cristae (Fig. 1F). We also observed individual cases of mitochondrial swelling in supporting cells (Fig. 1G). To objectively assess the state of the mitochondria, we used stained the OE for functionally active mitochondria with a fluorescent reagent (MitoTracker Orange) that selectively binds only native mitochondria with a conserved membrane potential. Compared with that in the control (Fig. 1H), the level of florescence in the mitochondria in the experimental fish was significantly lower (Fig. 1I). This fact was further confirmed by a comparative analysis of the volume occupied by the fluorescent signal in the mitochondria in OE cells of the control and experimental fish (Fig. 1J). The plot shows that the volume occupied by the mitochondria decreased by a factor of 6.2 (р_u_ ≤ 0.05) between the experimental and control fish.

**Figure 2:**
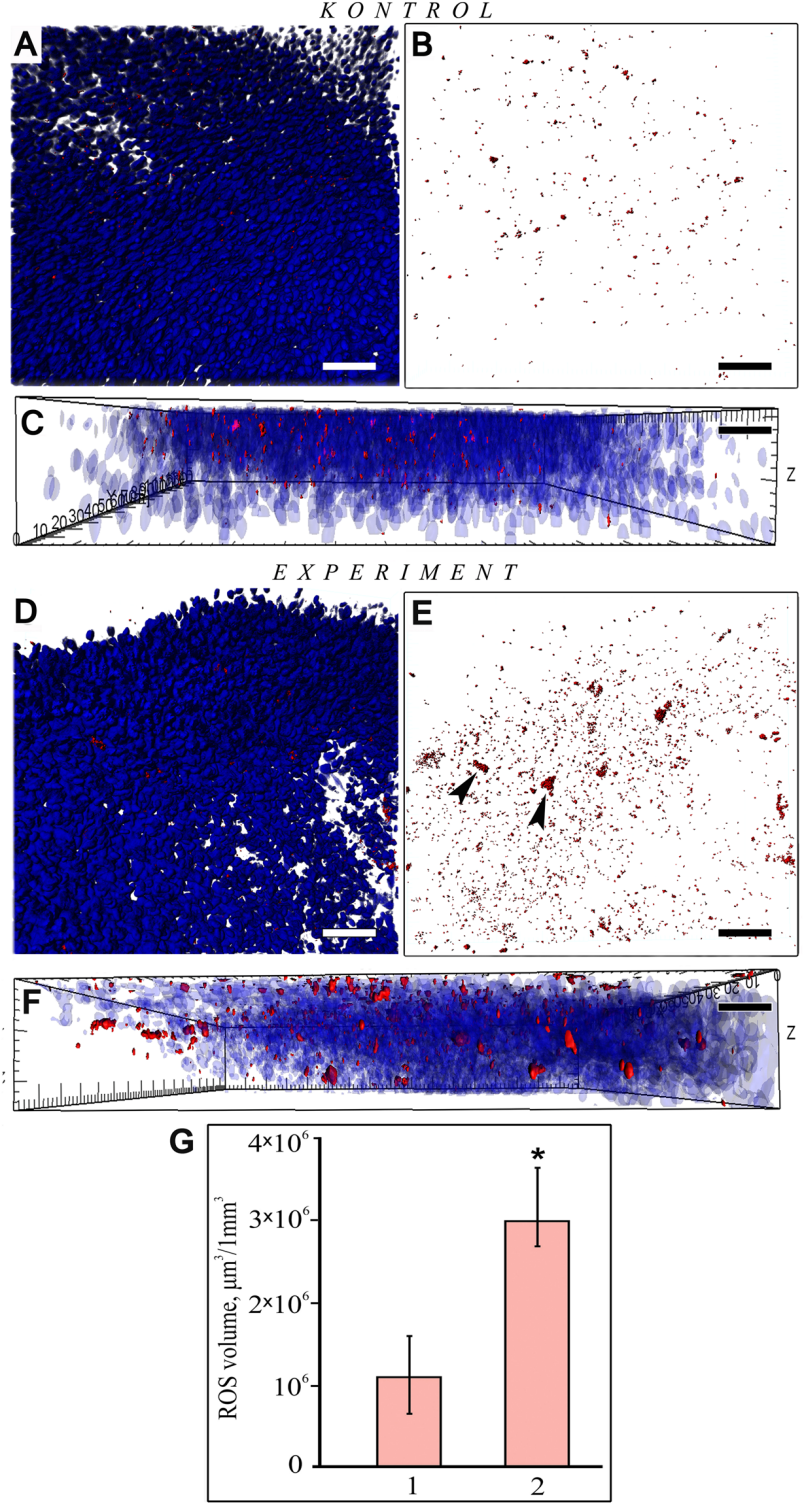
Features of ROS production in the ОЕ of *P. knerii* in the control group and after prolonged periodic odorant stimulation. (**А**‒**C**) A fragment of the OE in the control fish (А, blue and red channels; В, red channel); (**C**) the profile in Fig. A; the cytochemical reaction products were distributed as a fine substance. (**D**‒**F)** A fragment of the ОЕ in the experimental fish (D, blue and red channels, E, red channel); (**F**) the profile in Fig. D, E; the cytochemical reaction products, except for the fine substances, mostly formed large clusters (indicated by arrows). Staining for ROS (CellROX deep red reagent, red) and nuclei (DAPI, blue); confocal microscopy, 3D reconstructions. (**G**) The volume occupied by the fluorescent signal of ROS in the control group (**1**) and after prolonged odorant chemostimulation (2). The graph shows the data obtained by the quantitative analysis of the Z-stacks; confocal microscopy). *p_u_ ≤ 0.05 compared with the control group. Scale bars: A‒F, 30 mkm.

It is known that increasing the concentration of Ca^2^^+^ in the cytoplasm increases the production of reactive oxygen species (ROS) and caspase-dependent apoptosis of neuronal cells^44^.Therefore, we investigated the intensity of ROS formation in the OE of the control and experimental fish using the CellROX deep red reagent, which forms fluorescent products after a covalent interaction with free radicals in the cell. In the control preparations, the ROS products appeared as fine deposits that fluoresced in the red spectrum and were spread evenly across the width of the OE (Fig. 2A–C). In the experimental fish, however, these products appeared as more concentrated individual clusters as well as fine deposits (Fig. 2,D–F). Comparative statistical processing of the Z-stacks of the images with ROS products suggests that, compared with that in the control, this indicator in the experimental fish increased by a factor of 3.5 (р_u_ ≤ 0.05) (Fig. 2G). We hypothesize that such an increase in ROS production causes oxidative stress in the cell, since we observe this process along with degenerative changes in the olfactory epithelium in comparison with the control.

**Figure 3:**
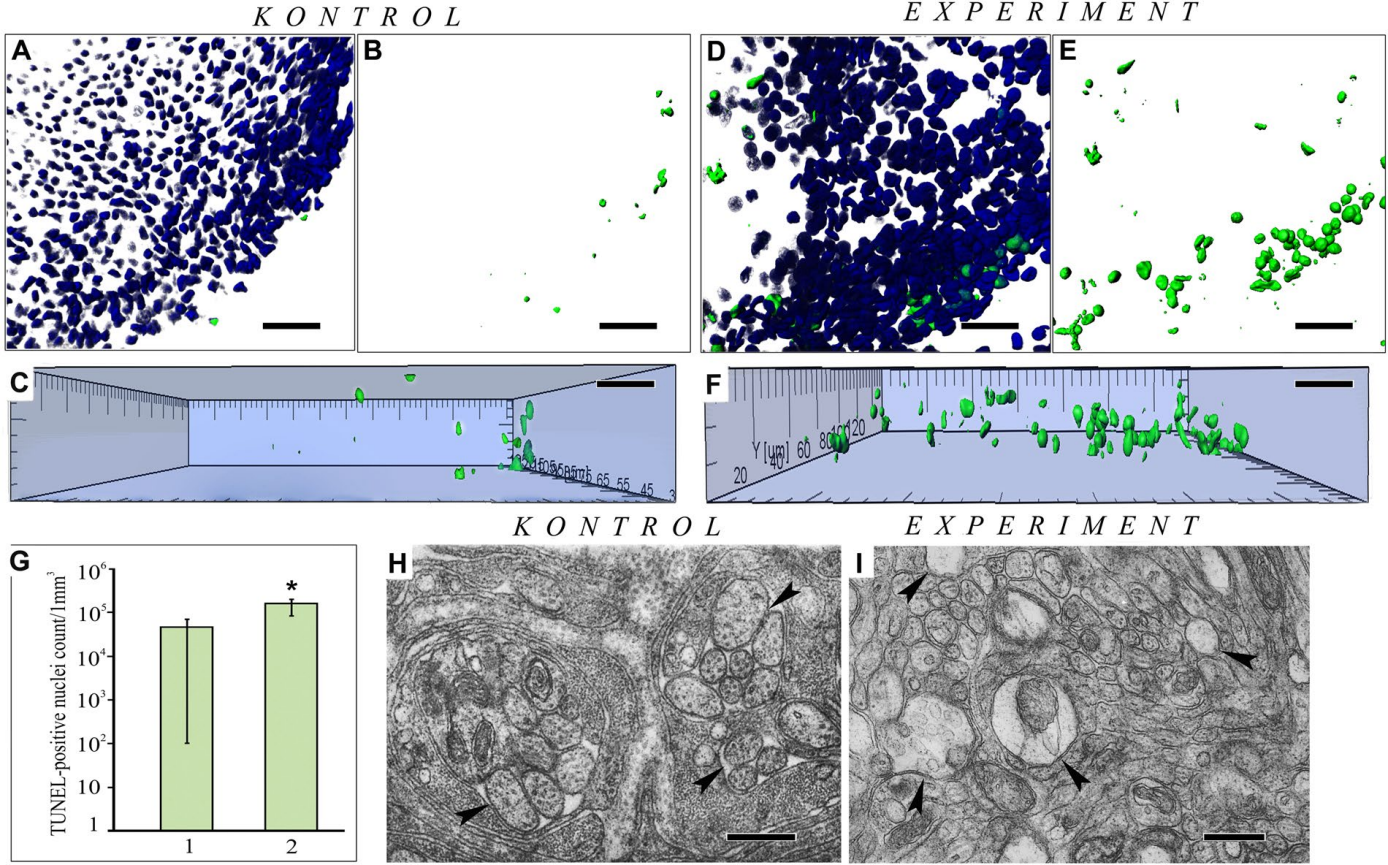
Neurodegenerative rearrangements in the OE of *P. knerii* in the control group and after prolonged periodic odorant stimulation. (**А**‒**C**) Features of PCD in the control group (А, blue and green channels; B,C green channel) and the experiment group (**D**‒**F**) (D, blue and green channels; E, F, green channel); (**C**) the profile in Fig. B; (**F**) the profile in Fig. E. In the Click-iT TUNEL assay, dead cell nuclei were stained with Alexa Fluor 488 (green), and the live ones were stained with Hoechst33342 (blue); confocal microscopy, 3D reconstructions. A substantial increase in the number of nuclei with fragmented DNA after prolonged odorant exposure. (**G**) The graph reflects the increase in PCD (1, control group; 2, experimental group). The graph shows the data obtained by the quantitative analysis of the Z-stacks; confocal microscopy). *p_u_ ≤ 0.05 compared with the control; (**H**) Cross section of normal axons (marked by arrows) OSNs in the basal region OE (control); (**I**) Cross section of swelled axons (marked by arrows) OSNs among normal axons in an experiment. Scale bars: A‒F, 20 mkm; H, I, 1 mkm.

To quantitatively assess PCD processes that may have been activated in the OE of the experimental fish during prolonged odorant stimulation, we used the TUNEL method^45^. This method allows cytochemical detection of DNA microfragmentation in cells during PCD. Figure 3A–C show a large OE fragment that exhibited scarce green-stained nuclei in cells with fragmented DNA in the control fish. By comparison, the experimental fish demonstrated a considerable increase in the number of cells undergoing PCD. These TUNEL-positive cells were distributed over the entire width of the epithelium, often forming more dense groups along the edge of the olfactory fold (Fig. 3D–F). The volume occupied by apoptotic nuclei was statistically analysed to show objectively that, compared with that in the control, the proportion of dead cells in the OE of the experimental fish increased by a factor of 4.5 (р_u_ ≤ 0.05) (Fig. 3G). Since degenerative changes were observed in both receptor and supporting cells in the experimental fish, as determined by electron microscopy, the plot can be supposed to display the amplification of PCD processes in both types of cells. Selective degenerative changes in axons in the basal OE layers also provided morphological evidence of the selective death of receptor cells under oxidative damage in intracellular structures. Compared to control (Fig. 3H), the axons in these OSNs were widened to various degrees, lacked neurofilaments, and showed a markedly lower density of cytoplasm (Fig. 3I). Axon swelling with destruction of the cytoskeleton components is one of the universal signs of degenerative changes in neurons not only in OE^46,47^, but also among neurons of the central nervous system^48^.

Based on the above evidence of significant degenerative changes in OE, we hypothesized that compensatory cell repair processes were activated in the OE of the experimental fish. To confirm this, we used bromodeoxyuridine (BrdU) labelling^49^ to compare the proliferative activity of cells in the OE of control and experimental fish subjected to prolonged odorant stimulation.

**Figure 4:**
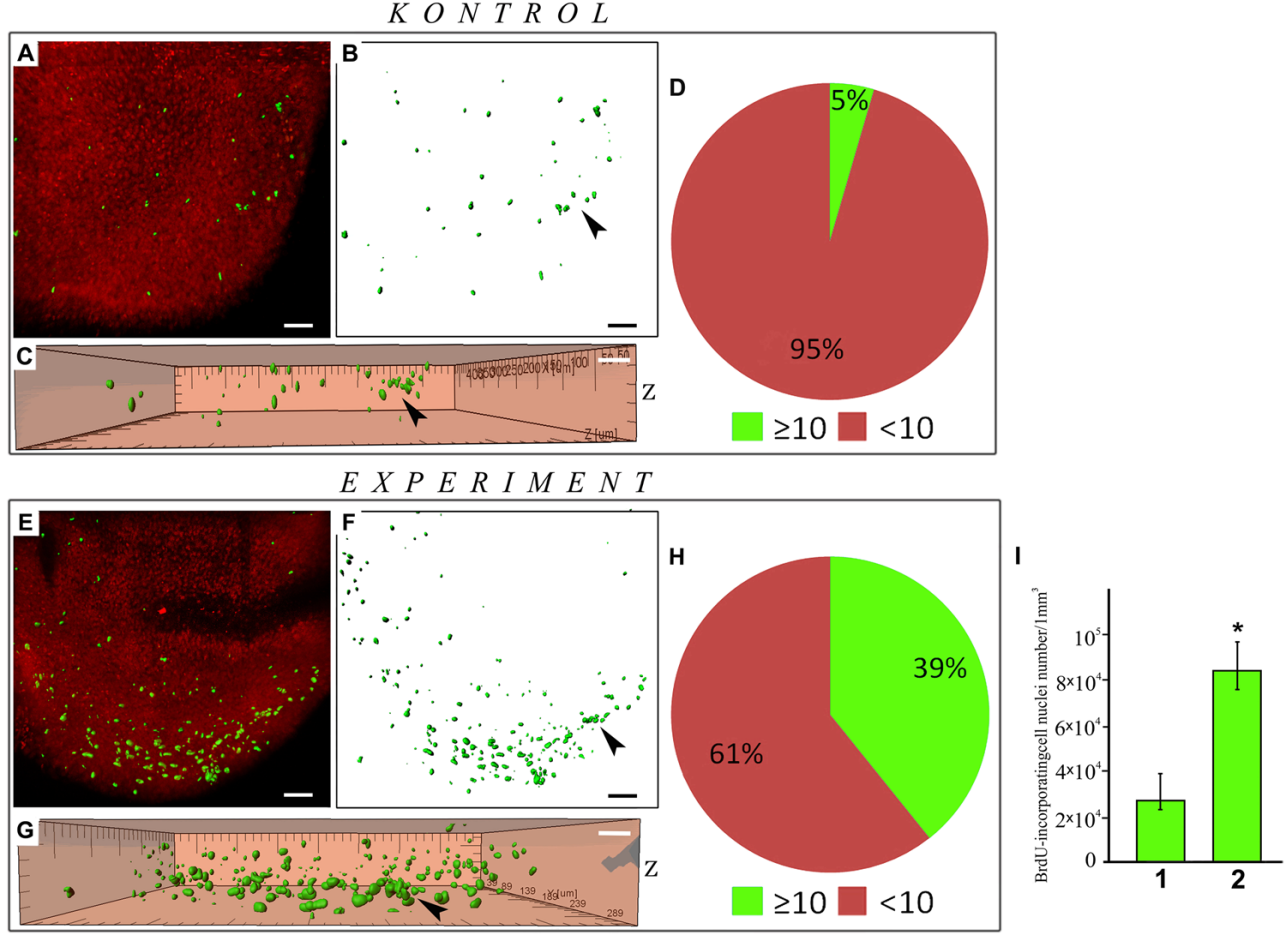
Enhancement of compensatory cell renewal in the olfactory folds of *P. knerii* in the control group and after prolonged odorant stimulation (12-h BrdU incubation). Selective staining for BrdU-incorporating nuclei with FITC anti-BrdU antibodies (green). Nondividing nuclei were stained with 7-aminoactinomycin (red); confocal microscopy, 3D reconstructions. (**А**‒**C**) BrdU-positive cells in the OE of the control fish (А, red and green channels; B, C, green channel); (**C**) the profile in Fig. A; (**D**) In the control fish, large cell clusters (10–20 cells; marked by arrows) accounted for 5% of the OE volume. (**E**‒**G**) BrdU-positive cells in the OE of the experiment group (E, red and green channels; F, G, green channel); (**G**) the profile in Fig. E. In the experimental fish (**H**), we observed an increase in the volume (up to 39%) of the OE zones with large clusters (10–20 cells; marked by arrows) of BrdU-incorporating cells (р <0.001). (**I**) The graph shows the enhancement of cell proliferation; 1, control group; 2, experimental group. The graph shows the data obtained by the quantitative analysis of the Z-stacks; confocal microscopy; *p_u_ ≤ 0.05 compared with the control group. Scale bars: A–C; E–G, 20 mkm.

In the control fish, BrdU-incorporating cells were usually located separately or in small clusters (up to 9 cells) or larger clusters (10–20 cells) (Fig. 4A–C; Supplementary video 1). Notably, the clusters composed of a small number of cells (9 or fewer) occupied 95% of the total volume of the tissue. The larger clusters (10–20 cells) of labelled nuclei accounted for only a small portion of the OE volume (5%) (Fig. 4D). The total number of label-incorporating cells in the control group was 5.06 ± 1.67 per 10^5^ µm.

By comparison, the OE of the fish subjected to prolonged odorant stimulation with a heterogeneous mixture of amino acids and peptides exhibited a considerable enhancement of cell proliferation processes (Fig. 4E–G; Supplementary video 2). Compared with that in the control, there was a substantial (by a factor of 7.8; р_u_ ≤ 0.05) increase in the number of areas populated by larger clusters of 10–20 BrdU-incorporating cells across the width of the OE. Simultaneously, the volume occupied by newly formed cells grouped into small clusters (up to 10 cells), decreased substantially (by a factor of 1.5; р_u_ ≤ 0.05) (Fig. 4H). The large clusters (10–20 cells) of BrdU-incorporating cells were located in different OE regions, but the highest concentration was in the periphery of the olfactory fold. It is characteristically that in that areas we found high amounts TUNEL-positive cells (Fig. 3D–F). Our calculations showed that the rate of cell proliferation processes in the OE of the experimental fish was higher than that in the control group by a factor of 3.4 (р_u_ ≤ 0.05) (Fig. 4I).

## Discussion

Our research shows that, in addition to the already known methods, the increased cell proliferation in OE of fish may also be caused by prolonged periodic exposure to natural water-soluble odorants. Given the monospecific nature of OSNs, a diverse set of these molecules can stimulate a large number of OSNs. As a result, swelling and a significant decrease in the functional activity of mitochondria, increased production of AFC, and in the end - PCD activation were observed in OE cells. We believe that these structural-functional cell rearrangements underlie compensatory activation of cell renewal processes in OE. How does the chain of cytochemical events, which leads to OE cell renewal, develop?

It is known that repetitive and prolonged exposure to odors causes an adaptive decrease in sensitivity, or *habituation*, in the olfactory system of animals^50,51^. This adaptation occurs at different levels of organization of the olfactory analyzer, along the entire path of sensory information processing^52^. The primary element in olfactory adaptation is the processes that occur in OSNs^53^.

It was found that the bonding of odorant molecules with OSN receptors activates adenylate cyclase, leading to an increase in the intracellular concentration of cyclic adenosine monophosphate (cAMP) and opening of cyclic nucleotide-gated (CNG) channels, through which Na^+^ and Са^2+^ ions penetrate into the cell^54,55^. This process serves as a trigger mechanism for depolarizing the cell. Moreover, in the cell activation process, phospholipase C together with diacylglycerol and inositol triphosphate also increase the intracellular concentration of Са^2+^ by means of intracellular depots^56^. A short-term increase in the Са^2+^ concentration opens Са^2+^-activated chloride (CAC) channels that amplify the CNG channel signal^57^.

At present, some data exist on the mechanisms underlying the short-term adaptation (STA) of OSNs to odorants. It was found that the STA can be caused by removal of intracellular Са^2+^ ^58^; by a decrease in the quantity of phosphodiesterase, which hydrolyzes cAMP and terminates its action^59^; by modulation of the cAMP-gated channel by Ca^2+^ feedback^60^; by cyclicity of the functioning of G-linked receptors (GPCR)^53^; and by other mechanisms. A decrease in the sensitivity of OSNs may occur in the presence of a prolonged stimulus as well as a short-term one. However, the processes that occur in OSNs exposed to prolonged odorant stimulation, either continuous or periodic, as of now remain unclear.

Electrophysiological studies conducted on cells of different types have shown that mitochondria are very sensitive to an excess of Ca^2+^ ions in the cytoplasm, especially under oxidative stress^61–63^. Calcium ions activate phospholipase A2, which is localized to the inner mitochondrial membrane, leading to phospholipid hydrolysis and a destabilization of the membrane. As a result of these processes, the membranes become more permeable to cations, which accumulate with phosphate in the matrix, leading to the accumulation of water inside the mitochondria which causes the mitochondria to swell, and interfere with oxidative phosphorylation processes^64^. Moreover, studies have revealed the possible formation of a large permeability transition pore in the inner mitochondrial membrane that may open in the presence of Ca^2+^, leading to the swelling of the mitochondria and to the release of water-soluble proapoptotic proteins such as cytochrome С, individual procaspases and AIF (apoptosis inducing factor), a direct cause of PCD, into the cytosol^65–71^. In turn, the enhancement of the processes of PCD can be a trigger for activating the processes of cell renewal in OE.

Thus, the developed method of noninvasive activation of cell reneval in OE allows one to generate an enriched pool of poorly differentiated neural cells as a source for transplantation. Cultivation followed by autological transplantation of these cells^72,73^ can be used in different fields of medicine. Presently, neural SC transplantation is applied to the development of methods for treating traumatic lesions^74,75^ of the nervous system, ischaemic brain damage^76^ and neurodegenerative processes^77–80^. The olfactory neural SC have neurocompetency and multipotency^81,82^, and their regenerative potential is shown in experiments with spinal cord regeneration in rats^73^.

In addition, it is known that dementia and Parkinson disease, which occurs in humans usually due to aging, as a result of an injury, or because of other factors, is accompanied by a decrease in the quantity of OSNs and olfactory dysfunction, leading to a disadaptation of the person^83–85^. In light of the data obtained, the proposed method for enhancing cell proliferation processes may bring about a substantial renewal of receptor cell populations and increase chemosensitivity in the peripheral part of the olfactory system. Thus, this approach can be used to develop methods for increasing the number of OSNs in patients with neurodegenerative diseases in which anosmia is present.

## Materials and Methods

### Animals

We studied neurodegenerative changes, PCD and compensatory renewal in the OE after the prolonged periodic stimulation with odorants of the stone sculpin *P. knerii* (Cottidae) (Dybowski, 1874) (Supplementary Fig. 1), an endemic representative of the Baikal ichthyofauna. *P. knerii* inhabits the entire shallow water zone of Lake Baikal to a depth of 200 m. The main prey items of *P. knerii* are amphipods, the larvae of amphibiotic insects, and zooplankton. Fish were caught in Southern Baikal by nets (depth 1.5–5.0 m) in spring (March-April) 2008–2018. The caught sculpins were kept in aquariums of the Baikal Museum with running water (4° С) from a depth of 400 m from Lake Baikal. The age of *P. knerii* was determined by the optical structure of the sagittal otoliths. The otoliths were extracted from the fishes’ skulls and prepared for study using standard methods^86,87^. The sagittal otoliths of *P. knerii* clearly show alternating semi-transparent and opaque rings corresponding to annual growth. The opaque rings were counted in the frontal plane from the nucleus to the proximal edge. Two lab workers independently counted the opaque rings in the frontal plane from the nucleus to the proximal edge using a passing light microscope (24–60×). When differences in age calculation appeared, the otoliths were re-examined by both lab workers until a consensus was reached. The morphological features, sex, and age of the *P. knerii* are given in Table 1.

**Table 1:** Morphometric features, age, and sex of the studied fishes.

Species	N	Length total, mm	Weight, g	Sex	Age (years)
*P. knerii*	50	98.6–116.2	14.2–21.7	♂	4 + –5+

To enable adaptation, the fish from the control and experimental groups (n = 25 in each group) were kept in aquariums (13 liters) with continuously renewed water for 7 days at 4 °C. Then, we stopped renewing the water and added a heterogeneous mixture of amino acids and peptides (0.0002% bacteriological grade Tryptone, a pancreatic digest of casein, VWR Life Science, AMRESCO, USA, cat no. Am-J859-0.25) to the aquariums with the experimental fish. The percentage composition of this mixture has been described previously^88^. Based on these data, a 0.0002% Tryptone solution contains amino acids as odorants in the concentration range from 6.60 × 10^−7^ to 1.02 × 10^−5^ M, which corresponds to the optimal range^22–27^.

The concentration of the substances was sustained for 15 days, except during the nighttime (lasting 12 h), when the fish were kept in a constant flow of running fresh water. Periodic renewal of fresh water is a necessary condition for the maintenance of Baikal fish. The control fish were kept in separate aquaria under the same conditions but were not exposed to the mixture of substances. When the experimental period passed, we extracted the olfactory rosettes from the control and experimental fish and prepared them for transmission and laser confocal microscopy (LCM).

In the OE of both the control and experimental fish, we cytochemically determined the volume occupied by ROS products, the volume of functionally active mitochondria, and the intensity of PCD with TUNEL^45^ and proliferative activity with BrdU^49^. Proliferative activity was assessed 12 h after intraperitoneal BrdU injection. The ultrastructural features of the preparations from the two groups were investigated using transmission electron microscopy. Both in control (n = 25) and in experiment (n = 25) for each technology LCM (3 methods) we used 5 fishes (10 olfactory organs), and for transmission electron microscopy we used 10 fishes (20 olfactory organs).

All experimental procedures were carried out in compliance with the guidelines of the EEC Directive 86/609 ЕЕС (1986) and approved by the Bioethics Committee of the Scientific Council, Faculty of Biology, Irkutsk State University, on November 30, 2007 (Resolution No. 3).

### Transmission electron microscopy

Olfactory rosettes were fixed with 2.5% glutaraldehyde solution (Sigma-Aldrich, USA) (prepared in 0.1 M phosphate buffer - PBS, pH 7.3) at 4 °C within 2 hours. After three washes in PBS, specimens were post-fixed in 2% OsO_4_ (Merck, Germany) in PBS for 12 hours, dehydrated in the ascending ethanol gradient with acetone, and encapsulated in resin (Araldite 502 Kit, SPI Supplies, USA). To estimate macro-anatomic features of fish olfactory rosettes of this preparations we made semi-thin sections (300–700 nm) with Ultracut R microtome (Leica, Germany). Stained with methylene blue (in a 1% aqueous solution, Sigma-Aldrich) that sections were investigated under an Axiovert 200 microscope (Carl Zeiss). Further, we obtained ultra-thin sections (70–80 nm) with the Ultracut R microtome (Leica, Germany). After staining with lead citrate, sections were analyzed with a transmission electron microscope Leo 906E at an accelerating voltage of 80 kV. Microscope images were taken with a MegaView II digital camera with the MegaVision software package (Soft Imaging System GmbH, Germany).

### Laser confocal microscopy

Since the olfactory organs of *P. knerii* are small (2–3 mm), their cytochemical features were studied entirely on the glass slide using an LSM 710 (Carl Zeiss) laser confocal microscope.

To stain functionally active mitochondria, olfactory rosettes were placed in medium 199 containing 200 nM MitoTracker Orange CMTMRos (ex/em 554⁄576 nm) (Thermo Fisher Scientific Inc., USA) for 30 min. The rosettes were then fixed in 2% paraformaldehyde for 15 min. Cell nuclei were stained with 0.5 μg/ml DAPI (ex/em 340/488 nm) (Sigma-Aldrich, USA) for 15 min. The preparations were embedded in the ProLong Gold Antifade reagent (Thermo Fisher Scientific Inc., USA) and studied using lasers: 405, 561 nm; filters: Ch1: 410–552, Ch2: 566–683.)

To analyse the reactive oxygen species production, the OE samples were incubated for 30 min in medium 199 with 5 μM CellROX Deep Red Reagent (ex/em 644/665 nm) (Thermo Fisher Scientific Inc., USA). The olfactory organs were then fixed in paraformaldehyde, stained with DAPI, and placed in ProLong Gold Antifade Reagent – same to the staining protocol for detection of functionally-active mitochondria. Preparations were studied using lasers: 405, 561 nm; filters: Ch1 410–522.

PCD was evaluated using the TUNEL method^45^ with a Click-iT TUNEL Alexa Fluor 488 Imaging Assay Reagent Set (Thermo Fisher Scientific Inc., USA), following the manufacturer’s recommendations. The process of material preparation included the following steps: fixation of the tissue in 4% paraformaldehyde, permeabilization in 0.25% Triton X-100, incubation in TdT (terminal deoxynucleotidyl transferase) reaction mixture with ethynyl-dUTP, click-reaction with Alexa Fluor 488 azide (ex/em 495/519 nm), and total nuclear staining with Hoechst 33342 (ex/em 350/460). Stained olfactory rosettes were placed on glass slides in a drop of ProLong Gold antifade reagent (Thermo Fisher Scientific Inc., USA). The preparations were then studied using lasers: channel 1, 405 nm, Ch1: 410–499; channel 2, 488 nm, Ch2: 499–725. Сell nuclei with fragmented DNA in the prepared slides fluoresced in the green region of the spectrum, and total nuclear DNA fluoresced in the blue region.

Proliferating cells in the OE were detected using the FITC BrdU Flow Kit (Becton, Dickinson and Company BD Biosciences, USA, Cat. No. 559619). This method is based on the incorporation of bromodeoxyuridine (BrdU) into DNA during the S-phase of the cell cycle^49^. The fish were labelled *in vivo* by intraperitoneal injection of BrdU in Dulbecco PBS (0.5 mg per 10 g body mass). Twelve hours after the BrdU injection, the olfactory rosettes were removed, fixed, permeabilized, and stained with FITC-conjugated anti-BrdU antibodies (to detect BrdU-positive cells) and 7-aminoactinomycin (to stain total nuclear DNA)^47^ according to the instructions of the FITC BrdU Flow Kit. After embedding in ProLong Gold antifade reagent (Thermo Fisher Scientific Inc., USA), the preparations were analysed using a lens Plan-Apochromat 20×/0.8 M27 and lasers: 561, 488 nm.

To assess the proliferative activity of OE cells, we obtained 15–20 Z-stacks from each olfactory rosette. The number of BrdU-incorporating cells in the tissue was calculated using Zen 2010 (Carl Zeiss) and Imaris Bitplane 7.2.3 software packages (Bitplane AG, Switzerland). In order to evaluate the spatial distribution of BrdU-positive cells, the Z-stacks of the images of olfactory folds were divided into separate smaller (1 × 10^5^ μm^3^) fragments. To estimate the volume of tissue in a Z-stack, we estimated the total volume of nuclei in tissue, occupied by the 7-AAD signal. In each volume of Z-stacks tissue, we counted the number of nuclei that included BrdU (based on binding of the FITC-conjugated antibody to BrdU). Based on the data obtained, we calculated the number of nuclei in 1 × 10^6^ μm^3^ of tissue for each Z-stack. Using a similar algorithm, we assessed the volume occupied by ROS products, the number of functionally active mitochondria, and PCD.

### Statistics

We analysed the data with non-parametric statistics, calculated the median, 25-, and 75-percentiles using the Statistica 10 software package. Intergroup differences were estimated using Mann–Whitney U and Chi square tests.

## Supplementary information

Supplementary Figure 1.

Supplementary Figure 2.

Supplementary video 1.

Supplementary video 2.
