# Supplementary material for: The Phenomenon of Compensatory Cell Proliferation in Olfactory Epithelium in Fish Caused by Prolonged Exposure to Natural Odorants
Source: Sci Rep. 2020 Jun 1;10:8908. doi: 10.1038/s41598-020-65854-9 (PMC7264137; doi:10.1038/s41598-020-65854-9)
Supplement: Supplementary file 1 — Supplementary Figure 1. [file 41598_2020_65854_MOESM1_ESM.pdf]

THE PHENOMENON OF COMPENSATORY CELL PROLIFERATION IN OLFACTORY EPITHELIUM IN FISH CAUSED BY PROLONGED EXPOSURE TO NATURAL ODORANTS

Igor V. Klimenkov[1,2*], Nikolay P. Sudakov[1], Mikhail V. Pastukhov[3] and Nikolay S. Kositsyn[4]

[1] Limnological Institute, Siberian Branch, Russian Academy of Sciences, 3 Ulan-Batorskaya St., Irkutsk, 664033 Russia
[2] Irkutsk State University, 1 Karl Marx St., Irkutsk, 664003 Russia
[3] Vinogradov Institute of Geochemistry, Siberian Branch, Russian Academy of Sciences, 1a Favorsky St., Irkutsk, 664033 Russia
[4] Institute of Higher Nervous Activity and Neurophysiology, Russian Academy of Sciences, 5a Butlerova St., Moscow, 117485 Russia

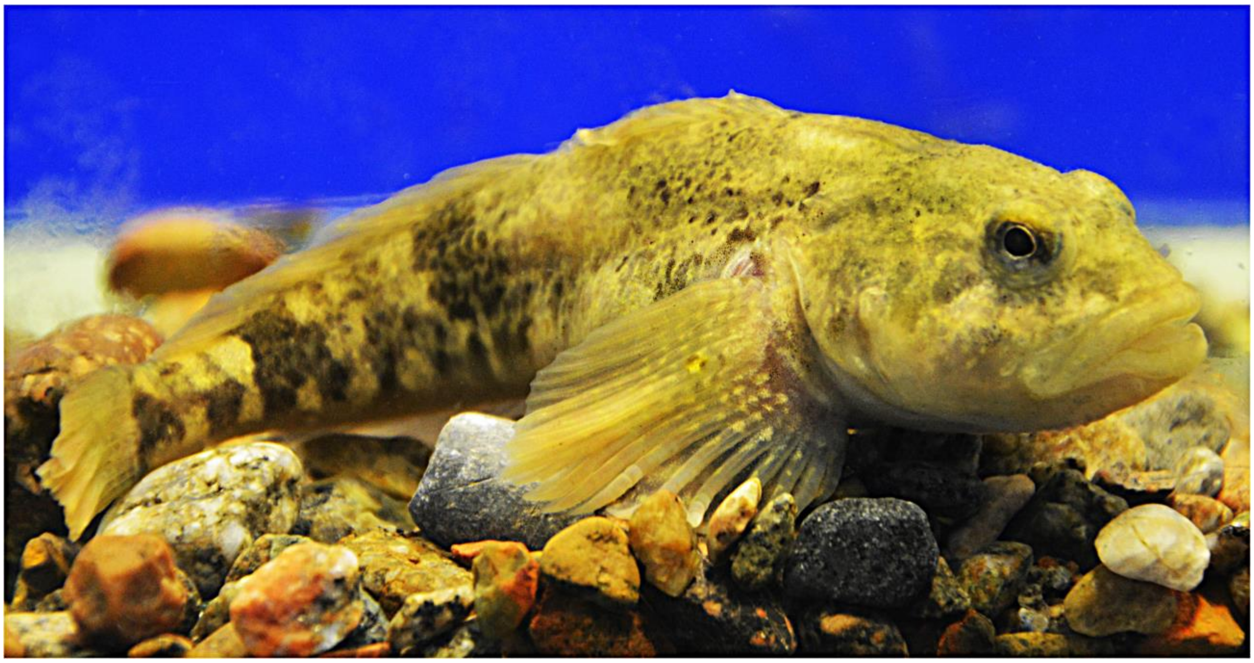

Supplementary Figure 1. *Paracottus knerii* (Dybowski, 1874) (Cottidae), endemic to Lake Baikal.
